# Gambling and screening for gambling problems in high school students before and after legal gambling age: associations with gender and gambling-related attitudes

**DOI:** 10.3389/fpubh.2026.1805134

**Published:** 2026-09-07

**Authors:** Anders Håkansson

**Affiliations:** 1 Department of Clinical Sciences Lund, Lund University, Lund, Sweden; 2 Skåne University Hospital, Psychiatry, Malmö, Sweden

**Keywords:** behavioral addiction, gambling, gambling disorder, high school, screening

## Abstract

**Introduction:**

Adolescence is a period associated with increased risk of gambling problems. Although strict age limits should apply in modern digital gambling platforms, gambling in minors remains a challenge. The present study aimed to study high school students’ gambling both before and after the legal gambling age, and to study associations between gambling-related attitudes and screening positive for problem gambling.

**Materials and methods:**

The study is based on a digital survey which was distributed by teachers to high school students in Lund, Sweden, in April/May, 2025 (*n* = 528). Self-reported history of gambling was reported for each form of gambling, and problem gambling was screened for using the three-item NODS-CLiP and combined with demographic data and gambling attitudes. Risk factors for screening positive for problem gambling were studied in multivariate logistic regression analyses, including gender and age, measures of gambling-related attitudes, and each separate gambling form.

**Results:**

A positive screen for problem gambling (NODS-CLiP >0) was seen in 16% at age 16, 15% at age 17, and 27% at age 18–19, in total among 31% of boys and 4% of girls. Gambling on online casino was reported by 41% if 18 + years and by 20% if below 18 years of age, and sports betting by 32 and 21% before and after 18 years of age, respectively. Screening positive for problem gambling was more common in case of parental acceptance of gambling, hiding gambling from parents, and liberal attitudes towards gambling policy, and in the multivariate analysis, problem gambling was significantly associated with online casino, sports betting, and gambling within videogames.

**Conclusion:**

In the present high school survey, gambling was very common, even before legal gambling age. Participants commonly screened positive for problem gambling, with a large risk increase in boys, and most strongly associated with online casino, sports betting, and gambling within videogames. Society-level policy interventions, as well as individual prevention targeting gambling attitudes, are called for.

## Introduction

Gambling for money can lead to the development of a severe addictive disorder (1), and can cause significant harm to affected users (2) and high financial costs to society (3). Despite common age restrictions, typically a legal gambling age in upper adolescence or young adulthood, gambling and gambling problems are common in the young (4, 5); in fact, younger age is associated with a higher prevalence of gambling problems than in middle-aged or older individuals (6).

Adolescents as well as adults are heavily exposed to gambling advertisements (7), and advertising is commonly promoting rapid, online-based gambling forms, which are also the same ones that are common in clinical settings (8). In recent years, high rates of early adolescent gambling have been demonstrated, although digital gambling and electronic authorization on gambling sites could theoretically facilitate the enforcement of age limits. In a recent high school survey in Sweden, already at 16 years of age, one out of ten high school students already reported having gambled for money on online casino sites, and one out of five adolescents of the same age reported having gambled for money on sports betting. In that prior study, 8 % screened positive for problem gambling at age 16, 6 % did so at aged 17, and 16 percent in the last grade of high school (when aged 18–19 years). In total, among high school students of all ages, a positive screen for problem gambling was seen in 13 percent of boys and in 5 % of girls (9).

High rates of adolescent gambling are a cause for concern, in particular in a novel gambling market with high availability of online gambling, while more traditional land-based gambling forms also remain, in parallel. However, the predominating role of highly accessible, online-based gambling forms today, as can be seen among patients seeking treatment for a gambling disorder (8), presents a novel situation which is not covered by older research on adolescent gambling (4, 10), and gambling problems need to be assessed both in relation to today’s gambling market, and in relation to relevant attitudes to gambling in adolescents and their parents. Therefore, the present study, in high school students both before and after the legal gambling age, aimed to analyze the reporting of life-time gambling on each of the most common gambling forms, to screen for gambling problems, and to study potential associations of gambling-related attitudes, and each of the gambling forms reported, with screening positive for gambling problems.

## Methods

The present study is a survey study conducted in school classes of two public high schools in the municipality of Lund, a city with around 130,000 inhabitants in southern Sweden. Headmasters of high schools were invited and the survey was distributed in school classes. These schools offer a broad range of regular high school curricula of the types which can prepare for post-high school university studies, including science, social sciences, technology, economy, and an artistic curriculum.

The study was approved by the Swedish Ethics Authority (file number 2025–00649-01). Surveys were designed such that no identifying information was collected, and it was clear to respondents that answers could not be traced back to any of the respondents. For the same reason of confidentiality, location and type of high school curriculum of the respondents were not collected. The survey was opened only in case the respondent actively confirmed her/his consent to participate.

In order to screen for gambling problems, this survey (see Supplementary material for English translation of the original Swedish questionnaire) included the NODS-CLiP tool (11), a three-item tool used for the screening of gambling problems. CLiP, the acronym for loss of Control, Lying, and Preoccupation, is well established as a brief screening tool, but not a diagnostic instrument. Also, the study applied selected variables from the Gambling Attitudes Measure (12), as well as parental attitudes corresponding to the items used in a previous high school study in the present setting (9). Dichotomized questions were asked about whether the respondent had ever gambled on each separate gambling form assessed (online casino, sports betting, horse race betting, poker, land-based card games, land-based electronic gambling machines, online bingo, and gambling for money within video games). Regarding the latter, the item in the survey was worded as “gambling for money within a video game, where you wager real money to either win real money or gain advantages within the game.” This variable, given its novel relevance in gambling behaviours in society (13), is increasingly being included among traditional gambling types in the present setting, such as in research surveys (9) and in clinical assessment tools (14), but in order to constitute an actual gambling behaviour, including in the present study, it explicitly addresses behaviours where real money is wagered and where the consequence of this behaviour is therefore a loss of money.

A total of 543 responses were opened, among whom 15 respondents did not provide consent to participate and for them, the survey was therefore closed thereafter. Thus, a total of 528 participants were included in the study. Fifty-two percent of respondents were male (*n* = 276), and 45 percent (*n* = 239) were female. Six individuals chose “other gender” and seven did not wish to report gender. These 13 individuals did not differ significantly from other participants with respect to age, screening for gambling problems, attitudes or history of gambling on each gambling form (two out of 12 participants, for whom screening data was available, screened positive for gambling problems). For this reason, individuals who did not endorse either male or female gender were excluded from statistical analyses, leaving gender to be dichotomized into male (54 percent, *n* = 276) and female (46 percent, *n* = 239) respondents. As sexual minorities have been described in the literature to be at elevated risk of behavioral addictions (15, 16), the final logistic regression analysis with a focus on the problem gambling screening was performed with a sensitivity analysis that excluded the gender variable but which included all individuals, including respondents who wished not to report gender or who reported another gender identity. Age was missing in no cases. Mean age in the dataset was 17.0 years (SD 0.87, median 17 years, inter-quartile range 16–18). Males were in majority in all age groups (Table 1). Full NODS-CLiP data, allowing for the screening for gambling problems, was missing for 18 individuals and available in 510 respondents. Missing data were not replaced, and therefore omitted from analyses.

**Table 1:** Gender distribution in each age group.

	Male, % (*n*)	Female, % (*n*)	All (*n*)
15–16 years	51 (77)	49 (74)	151
17 years	55 (120)	45 (99)	219
18 years	54 (62)	46 (53)	115
19 years	57 (17)	43 (13)	30
All ages	54 (276)	46 (239)	515

Statistical calculations included Chi-square tests for dichotomized group comparisons, including the linear-by-linear Chi-square test for the age variable and for parental acceptance of gambling. Statistical significance was considered if *p* < 0.05. Chi-square values, degrees of freedom (df), and level of significance (*p* value) are reported. Thereafter, two logistic regression analyses were conducted, assessing correlates of a positive screen for problem gambling. In the first analysis, gender, age, and gambling-attitude-related variables were included. Thereafter, each of the included gambling forms were added into the model. Results were reported as odds ratios (OR) with 95-percent confidence intervals. In order to assess the risk of multicollinearity in variables entered into the logistic regression, a correlation matrix was run for each of these variables pair-wise. The highest Pearson correlations seen were for online casino and sports betting (*r* = 0.65), online casino and poker (*r* = 0.56), online casino and gender (*r* = 0.48), poker and sports betting (*r* = 0.48), card games and online casino (*r* = 0.48), card games and sports betting (*r* = 0.48), gambling within videogames and online casino (*r* = 0.43), gambling within videogames and poker (*n* = 0.43), and gambling within videogames and gender (*r* = 0.41), which were all considered to be acceptable for inclusion (below a suggested cut-off value of *r* = 0.70) into a logistic regression model (17).

## Results

### Experience of gambling

Rates of lifetime experience of gambling, on each gambling form, are displayed in Table 2, for males and females, respectively. Self-reported gambling on sports betting (Chi-square 13.44, df 4, *p* < 0.01) and online casino (28.81, 4, *p* < 0.001) were more commonly reported with increasing age (Chi-square linear-by-linear), whereas this association was not seen for other gambling forms. Having gambled on online casino was reported by 20 percent of participants aged below the legal gambling age of 18 years, and by 41 percent of those aged 18–19 years (24.00, 1, *p* < 0.001). Sports betting, likewise, was reported by 21 percent below 18 years of age, and by 32 percent of 18–19-year-olds (8.08, 1, *p* < 0.01). Horse race betting was reported by six and 2 % of those younger than 18 and 18–19 years (3.05, 1, *p* = 0.08), respectively, and poker was reported by 14 percent before 18 years of age, and by 20 percent aged 18–19, respectively (3.38, 1, *p* = 0.07). In the corresponding age groups, electronic gambling machines were reported by seven and 10 %, respectively (1.25, 1, *p* = 0.26), card games by 34 and 33 percent, respectively (0.07, 1, *p* = 0.79), online bingo by ten and 9 %, respectively (0.07, 1, *p* = 0.79), and gambling within video games by 25 and 20 percent, respectively (1.37, 1, *p* = 0.24).

**Table 2:** Self-reported history of ever having gambled on each of the specific gambling forms included.

Ever gambled, % (*n*)	All	Male	Female	Chi-square for gender difference (Chi-square value, df, *p*)
Online casino	26 (135)	46 (126)	4 (9)	116.18, 1, <0.001
Sports betting	24 (123)	39 (109)	6 (14)	79.71, 1, <0.001
Horse race betting	5 (24)	7 (18)	3 (6)	4.64, 1, 0.03
Poker	15 (79)	27 (74)	2 (5)	60.27, 1, <0.001
Electronic gambling machines, physical	8 (39)	9 (26)	5 (13)	2.90, 1, 0.09
Cards, physical	34 (175)	51 (142)	14 (33)	80.90, 1, <0.001
Online bingo	10 (49)	9 (26)	10 (23)	0.01, 1, 0.94
Gambling within videogames	23 (121)	40 (110)	5 (11)	88.56, 1, <0.001

High school students aged 15–19. % (*n*).

### Screening positive for problem gambling

A positive screen for gambling problem was seen in 31 percent (*n* = 82) of males and 4 % (*n* = 10) of females (57.24, 1, *p* < 0.001), thus in total, in 18 percent (*n* = 92) of all respondents (498 individuals for whom both gender and problem criteria were available). The risk of screening positive for problem gambling increased with increasing age (13.82, 4, *p* = 0.04, Chi-square linear-by-linear), with 27 percent of 18, 19-year-olds who screened positive for problem gambling, compared to 16 percent of those aged 15–16, and 15 percent of those aged 17 years (in total 15 percent of all respondents aged younger than 18).

Within the NODS-CLiP screening tool, 10% of respondents endorsed the tolerance criterion (*n* = 51, answers missing in four cases), 12 percent endorsed the criterion of attempts to quit or cut down (*n* = 64, 16 missing answers), and 8% endorsed the criterion of lying (*n* = 40, five missing answers). In total, 10% endorsed one criterion (*n* = 49), 4% endorsed two criteria (*n* = 23), and 4% endorsed all three criteria (*n* = 20), whereas 79 percent did not endorse any of the criteria (*n* = 406, 17 missing answers).

The subjective experience of ever having felt a gambling problem was endorsed by 5% (*n* = 25, five missing answers, 9 percent of males and 0 percent of females, 22.82, 1, *p* < 0.001), and 7% (*n* = 38, five missing answers, 13 percent of males and 1 percent of females, 28.03,1, *p* < 0.001) reported that somebody else had felt the respondent had a gambling problem.

All gambling forms were more likely to be reported by those who screened positive for gambling problems than among others; online casino was reported by 80 percent of those who screened positive vs. 15 percent of those who did not (162.40, 1, *p* < 0.001), sports betting by 73 vs. 14 percent (140.54, 1, *p* < 0.001), horse race betting by 13 vs. 3 percent (16.64, 1, *p* < 0.001), poker by 51 vs. 8 percent (104.90, 1, *p* < 0.001), electronic gambling machines by 17 vs. 6 percent (14.29, 1, *p* < 0.001), cards by 71 vs. 27 percent (63.31, 1, *p* < 0.001), online bingo by 18 vs. 8 percent (9.49, 1, *p* < 0.01), and gambling within video games by 64 vs. 15 percent (98.89, 1, *p* < 0.001).

### Respondents’ and parents’ gambling attitudes

Two percent of respondents reported that for the parents, it was “completely ok” if the respondent was gambling, 6 % that that it was “quite ok,” 20 percent neither, 29 percent that it was “not quite ok” and 41 percent that it was “absolutely not ok,” whereas six participants did not respond. Respondents reported that they were hiding their gambling from their parents “only slightly” (6 %), “to some extent” (2 %), “quite a lot” (3 %), “a lot” (3 %), or “not at all” (14 percent), and 71 percent reported to be ‘not gambling’ (two missing answers).

Eighteen percent answered that gambling is “morally wrong,” 48 percent that it was not, and 34 were uncertain (one missing answer). Twenty-two percent believed that all gambling forms should be legal, 71 percent that some forms should be legal and some should be illegal, and 7 % believed all gambling should be illegal (one missing answer). Regarding the availability of gambling in Sweden, 43 percent believed gambling availability is too high, 48 percent that availability should be as it is today, and 8 % that gambling should be more available (six missing answers).

Screening positive for gambling problems was associated with a higher degree of parental acceptance towards gambling (22.89, 1, *p* < 0.001). Respondents who screened positive were more likely to report hiding gambling from their parents (70 vs. 34 percent, 19.74, 1, *p* < 0.001, individuals only included if they did not report that they were not gambling), more likely to report that all gambling should be legal (51 vs. 25 percent, 25.23, 1, *p* < 0.001), and more likely to report that gambling availability should be as it is or increase (74 vs. 53 percent, 13.43, 1, *p* < 0.001), but were not significantly less likely to report that gambling was morally wrong (13 vs. 19 percent, 1.79, 1, *p* = 0.18).

### Factors associated with screening positive for gambling problems

In a logistic regression analysis, including age, gender, and respondents’ and parents’ gambling-related attitudes, a positive screen for problem gambling was significantly associated with male gender, older age, allowing parental attitudes towards gambling, and with the attitude that all gambling should be legal, but not significantly associated with the attitude that gambling should be as available as it is, or more, or with the statement that gambling is morally wrong. In a second step, adding all gambling forms into the model, screening positive for gambling problems did not remain significantly associated with gender, age, or with gambling attitudes, but was associated with online casino, sports betting, and with gambling within videogames, but not with any other separate gambling form (see Table 3 for full regression analyses).

**Table 3:** Correlates of screening positive for gambling problems.

	OR (95 percent confidence interval)	*p*-value	OR (95 percent confidence interval)	*p*-value
Male gender	7.28 (3.58–14.80)	<0.001	1.07 (0.41–2.77)	0.89
Older age	1.33 (1.00–1.77)	0.05	1.16 (0.81–1.67)	0.41
Allowing parental gambling attitudes	1.35 (1.06–1.70)	0.01	1.11 (0.82–1.51)	0.48
All gambling should be legal	1.90 (1.12–3.22)	0.02	1.71 (0.88–3.32)	0.11
Gambling available as it is or more	1.62 (0.91–2.88)	0.10	1.24 (0.60–2.57)	0.57
Gambling is morally wrong	1.17 (0.54–2.52)	0.69	1.61 (0.61–4.27)	0.34
Online casino			5.23 (2.25–12.13)	<0.001
Sports betting			3.24 (1.52–6.89)	<0.01
Gambling within videogames			3.48 (1.78–6.82)	<0.001
Horse race betting			0.69 (0.21–2.27)	0.54
Poker			1.71 (0.81–3.63)	0.16
Electronic gambling machines, physical			0.85 (0.29–2.43)	0.76
Cards, physical			1.37 (0.67–2.81)	0.39
Online bingo			1.88 (0.71–4.99)	0.20

Logistic regression analyses in two steps.

## Conclusion

In the present high school survey study, in line with previous studies in similar high school contexts, gambling was common, both before and after the legal gambling age, and even for gambling forms for which a strict age restriction is typically applied in legal gambling operators. In the present study, it was also evident that some gambling forms were more closely related to a positive screen for problem gambling than others. In particular, online casino gambling and sports betting, both known to be common in treatment settings among adults (8), were associated with screening positive for problem gambling, but this was also the case for gambling within videogames. Also, compared to a previous comparable high school study in a corresponding geographical setting, rates of gambling, and rates of screening positive for gambling problems, are now markedly increased. Gender differences are large, with a positive screen detected among almost one third of boys, and rates of ever using some gambling forms reached almost half of male respondents. As in previous research, more allowing individual and parental attitudes towards gambling were associated with screening positive for gambling problems.

In the previous survey study from a similar setting, i.e., in high school students from a range of schools in the same region in southern Sweden, the gender gap in the problem gambling screening appeared to be narrowing, with 13 percent of boys and 5% of girls who screened positive (9). In that study, this gender gap appeared to be smaller than in somewhat older data from a corresponding age group and, again, in the same geographical setting (10). Also, in a general population study of 18-year olds where the gender gap was large, with rates of problem or pathological gambling reaching almost 6% in boys and 0.4 percent in girls, and in adolescents aged 18 years, more than 15 percent of boys (and only around 1% of girls) fulfilled criteria of at least “risky gambling,” a degree of problems which may be comparable to the measure used in the present study (18). In the present study, a positive screen for gambling problem was seen in 31 percent of boys and 4% of girls, thereby also representing a large gap. It cannot be concluded whether this reflects an ongoing trend towards increasing gambling in young boys, compared to the prior but relatively recent study, or whether characteristics of the included study samples may differ. However, altogether, most research from this setting indicates that male gender is an even stronger gambling-related risk factor in the young, than in older age groups (4, 10, 18), and the present study confirms this.

High rates of gambling for money during high school, both after and before legal gambling age, calls for preventive efforts addressing adolescents. In this study, screening positive for problem gambling was strongly linked to parental acceptance of gambling and paradoxically linked to hiding gambling from parents, and also associated with liberal attitudes towards gambling policy. Adolescents who screened positive appeared to be more likely to report that all gambling should be legal, that availability of gambling in Sweden should either remain or increase, and that they were less likely to report that gambling was morally wrong. Thus, the association between liberal gambling attitudes and a positive problem screen was, in this study, in line with the findings from our previous high school study in the same setting (9). Thus, it can be argued that gambling-related attitudes could be one of the targets of preventive actions. Also, parental attitudes towards gambling may facilitate gambling in the young and can also be a target for public health interventions. In the previous study, having gambled together with parents was also associated with screening positive for problems, a variable that was not assessed in the present study.

Although there is emerging scientific support favouring prevention against gambling problems in the young, research is scarce and studies are needed which test different methods in order to prevent early onset of gambling problems (19). Over and above individual-level preventive work, such as interventions aiming to change gambling attitudes or adolescents’ proneness to gambling, there is reason to highlight the society-level perspective of gambling regulations, policies for advertising that may reach young individuals above or before the legal gambling age, and enforcement of technical solutions against under-age online gambling, such as through improved identification requirements on gambling web sites.

Gambling within videogames was commonly reported, and one of the gambling forms which remained associated with a positive screen for problem gambling in the multi-variate analysis. This gambling form represents an emerging issue and can be broadly defined, in the context of extensive video gaming habits which are very widespread in adolescents in approximately these age groups, in particular among boys (20). Results call for a focus on gambling elements within videogames in future research and preventive work against problem gambling in the young. Likewise, some of the more common gambling forms were the ones which are most common in treatment-seeking clients with a gambling disorder, i.e., highly accessible and online-based casino games and online sports betting (8). Online casino and sports betting, along with gambling within videogames, remained significantly associated with the gambling problem screening in the multi-variate analysis. The highly diverse addictive potential of different forms of gambling has been highlighted and summarized in several previous studies, and casino games and electronic gambling machines typically stand out as being more addictive (21). Online casino and sports betting are the gambling forms which are, by far, the most common in patients seeking gambling disorder treatment (8). Casino games in particular have been pointed out in a previous Swedish population survey among the forms of gambling most closely linked to gambling problems in the population, along with other chance-based, rapid games such as electronic gambling machines and bingo, although, in that population survey, sports betting had an intermediate position in relation to gambling problems, between casino games and lotteries which demonstrated substantially lower addictive potential. While land-based electronic gambling machines have been pointed out as linked to gambling problems in the population (22), these have declined substantially as a gambling problem reported in clinical settings among adults (8), and are–due to age limits at the physical gambling venues–less likely to be used by minors. For these reasons, it can be seen as unsurprising that online casino and sports betting are the most strongly related to gambling problems also in adolescents. While physical card games remain common, in particular among males (9), the present study again highlights the role of online gambling in adolescents and that the form of gambling typically considered to have the most outstanding addiction potential today, online casino (9, 23), was indeed reported by close than half of male respondents in the study.

The present study used a brief screening tool for potential gambling problems. As the choice of a brief instrument was based on the need to reduce the extent of the survey sent out to high school students, in order to avoid high rates of attrition, it can be argued that the endorsement of screening criteria may not always reflect a more serious or clinical gambling problem. The diagnostic level of problems in this sample is therefore hard to estimate. According to findings from a corresponding adolescent population, those who fulfil the criteria of a gambling-related diagnosis represent only a minority of those who display any hazardous or problematic gambling behaviour; especially among boys, in that study, risk or problem gambling was around five times more common than pathological gambling (18). Thus, a large proportion of the 31 percent of boys and 4 % of girls who screened positive for a gambling problem, would not be considered to have a gambling-related diagnosis in a clinical assessment. Still, given the risks associated with early gambling onset and its financial and social consequences, findings are still of relevance to the problem burden of young people with respect to gambling, and importantly, the reporting of rapid, chance-based or skills-based gambling forms known to confer a high risk of addiction, even before they should be legally accessed, is a cause for concern.

Today’s gambling pattern in many settings, in particular in the present setting, could be expected to complicate gambling in individuals who are younger than the legal gambling age, as the electronic identification upon entry on a gambling site should facilitate age limits. In Sweden, a vast majority of patients seeking treatment for a gambling disorder report their gambling problem to be related to the online gambling modality. It is unknown how adolescents below 18 years of age, such as in the present study, access gambling for money even in a highly digitalized gambling market where high-security authentication is standard. The risk of gambling access before legal gambling age can be seen in parallel with the challenge of limiting gambling on overseas operators which do not adhere to the policies enforced within the domestic market. It is well documented that for example, individuals who self-exclude from gambling because of a gambling problem may be at risk of relapsing despite this, either on overseas gambling sites, or through gambling on another person’s identify (8). It can be suspected that a similar pattern may be seen for minors who access gambling sites from which they should legally be excluded, but from the data studied here, we cannot conclude to what extent each form of irregular gambling habits that explain minors’ access to online gambling here. Likewise, not all gambling reported here is carried out through regulated gambling operators; land-based card games, and possibly some poker gambling, is likely to be carried out in entirely private settings, and regarding gambling for money within video games, despite its likely relevance to the overall field of problem gambling (13), legal regulations of this gambling behaviour is known to be highly challenging (24).

This study has limitations. First, the study included only high school curricula which can prepare students for a university education, instead of the more practically oriented high school programs. As gambling is traditionally reported to be associated with a lower rather than higher level of schooling (25, 26), including an association between lower school achievements and gambling problems in the young (27), this limitation would imply that despite the high rates of gambling seen here and the high percentage of students screening positive for problems, gambling could theoretically even be under-reported in this study compared to more practically oriented high school programs. This, however, cannot be established and remains a limitation of this research. Again, it demonstrates a screening for gambling problems rather than a diagnostic procedure, in line with the need to shorten the content of an online survey tool, and it also examines respondents’ reporting of any gambling experience on each gambling form, rather than frequencies, financial amounts or other measures of gambling severity. Likewise, as the survey guaranteed full confidentiality, even such that a survey response could not be traced back to a specific user, the rates of attrition cannot be established. However, among respondents who opened the electronic survey, very few denied study participation and closed down the survey tool. While anonymous study procedures are key in the detection of underage gambling as well as many other addictive behaviours in the young, future research designs should attempt to follow underage gambling even in designs where attrition can be accounted for and where refusal to participate can be detected and quantified.

Also, given the full confidentiality approach, response rates cannot be calculated and this also represents a limitation. In a sensitivity analysis, aiming to include groups of responses believed to represent school classes with a high response rate, it was assumed that responses from more than 20 students approximately at the same time would correspond to a school class, as nearly all school classes at these two high schools have 28–32 students (personal communication, municipality of Lund). Responses could be grouped into 15 such groups (assumingly 15 classes), including a total of 353 responses. When including only these responses, 29 percent of males and 5 % of females screened positive for gambling problems, i.e., close to the full analysis. In the final logistic regression, a positive problem screen remained associated (with comparable odds ratios) with sports betting, online casino, and gambling within videogames. Thus, in a sensitivity analysis believed to include school classes with a high response rate, results remained similar to the whole sample. Future studies should follow high school students addressed by the offer to participate and register their participation or non-participation, although while respecting the need for confidentiality and thereby the difficulty in following identified individuals in a study on a sensitive topic such as the research topic addressed here.

Likewise, a sensitivity analysis was carried out where all individuals were included, including respondents who endorsed another gender identity or who did not wish to answer the gender item (13 individuals). When including them, and omitting gender from the logistic regression analysis, the final analysis identified the same risk factors for screening positive for gambling problems, with very similar odds ratios (4.98 instead of 5.23 for online casino, 3.31 instead of 3.24 for sports betting, and 3.60 instead of 3.48 for gambling within videogames). As the rates of gambling history, screening for gambling problems or gambling-related attitudes in the 13 individuals with other or missing gender did not differ from the rest of the sample, it was concluded that the choice to analyze, statistically in regression analyses, women and men, did not alter the picture of risk factors and gambling patterns described in the paper. Future research, however, should attempt to include larger study samples that can allow for sub-group analyses of individuals with a non-binary gender identification, for example, as sexual minorities have been described to be at some increased risk of behavioral addictions (15, 16).

In addition, associations discussed in the present study cannot be expected to represent causal associations; for example, the link between screening positive for gambling problems and endorsing liberal gambling attitudes might underscore that such attitudes may facilitate adolescent gambling, but the inverse causality cannot be ruled out. However, regardless of the actual causality in such an association, it still can be considered relevant to include discussions around gambling-related attitudes in future preventive interventions, as they are likely to play a role either as a cause or a consequence, or both, in relation to risky gambling habits. Regarding the forms of gambling included in the present study, these were believed to cover the vast majority of gambling behaviours reported in previous clinical settings in Sweden (8), although novel variations of previously established gambling patterns, such as fantasy sports as a variation of regular sports betting (28), may not necessarily be referred to by respondents as sports betting or any of the other options listed in the study. Thus, in a longer survey format in future studies, it would be fruitful to include a larger and more detailed list of diverse forms of gambling for money.

To conclude, in the present high school survey study, as in previous similar surveys, a history of gambling is commonly reported, in ages both below and above the legal gambling age. Gambling forms which typically are seen in treatment-seeking adults, and which are believed to confer a high addiction potential, are commonly reported by adolescents already in the first degree of high school, to a markedly higher extent in boys, but gambling within videogames was also associated with the endorsing of screening items for gambling problems. Findings call for prevention and early interventions when gambling is detected in adolescents. From the findings presented here, positive attitudes towards gambling, both in adolescents and in their parents, appear to be associated with a positive problem gambling screen in the young, and should be addressed in such interventions, as well as legal interventions targeting age restrictions and technical gambling access in minors.

## Data Availability

The raw data supporting the conclusions of this article will be made available by the authors, without undue reservation.
