# Supplementary material for: Gambling and screening for gambling problems in high school students before and after legal gambling age: associations with gender and gambling-related attitudes
Source: Front Public Health. 2026 Sep 7;14:1805134. doi: 10.3389/fpubh.2026.1805134 (PMC13590350; doi:10.3389/fpubh.2026.1805134)
Supplement: Supplementary file 1 [file Data_Sheet_1.PDF]

Are you…?

☐ Female

☐ Male

☐ Another gender identity

☐ Prefer not to answer

How old are you?

☐ 15

☐ 16

☐ 17

☐ 18

☐ 19

Some questions about gambling:

Have you ever, at any point in your life, played the following forms of gambling?

Online casino games (online casino, slots, roulette, blackjack, etc. online)?
☐ Yes ☐ No

Sports betting (in a shop or online)?
☐ Yes ☐ No

Horse betting (at a betting shop/racetrack or online)?
☐ Yes ☐ No

Online poker
☐ Yes ☐ No

Slot machines ('Vegas') in bars, restaurants, and similar venues
☐ Yes ☐ No

Physical card games money
☐ Yes ☐ No

Online bingo
☐ Yes ☐ No

Gambling for money within a video game, where you wager real money to either win real money or gain advantages within the game?

☐ Yes ☐ No

Have you ever had a period of two weeks or more when you spent a lot of time thinking about your gambling or planning future bets or wagers?

☐ Yes ☐ No

Have you ever tried to stop gambling, cut down, or control your gambling?

☐ Yes ☐ No

Have you ever lied to family members, friends, or others about how much you gamble (for money) and how much money you have lost gambling?

☐ Yes ☐ No

Have you ever personally felt that you had problems with your gambling?

☐ Yes ☐ No

Has anyone else around you thought that you have problems with gambling?

☐ Yes ☐ No

What do your parents think about your gambling habits? Select the response that best fits.

My parents are OK with me gambling for money.

☐ No, they are not OK with it at all

☐ No, they are not particularly OK with it

☐ Neither agree nor disagree

☐ They are fairly OK with it

☐ They are completely OK with it

Do you hide your gambling habits from your parents?

☐ Very much

☐ Quite a bit

☐ Somewhat
☐ Just a little
☐ Not at all

☐ I do not gamble

Some questions about your opinions on gambling:

Do you think gambling is morally wrong?

☐ No
☐ Yes
☐ Unsure / Don't know

Which statement best reflects your views on whether gambling should be legal?

☐ All forms of gambling should be legal
☐ All forms of gambling should be illegal
☐ Some forms of gambling should be legal and some forms should be illegal

What do you think about the availability of gambling opportunities in Sweden?

☐ Gambling is too accessible
☐ Gambling should be more accessible
☐ Gambling is appropriately accessible as it is
